# Heterogeneous ribonuclear protein E2 (hnRNP E2) is associated with TDP-43-immunoreactive neurites in Semantic Dementia but not with other TDP-43 pathological subtypes of Frontotemporal Lobar Degeneration

**DOI:** 10.1186/s40478-017-0454-4

**Published:** 2017-06-30

**Authors:** Yvonne S. Davidson, Andrew C. Robinson, Louis Flood, Sara Rollinson, Bridget C. Benson, Yasmine T. Asi, Anna Richardson, Matthew Jones, Julie S. Snowden, Stuart Pickering-Brown, Tammaryn Lashley, David M. A. Mann

**Affiliations:** 1 Division of Neuroscience and Experimental Psychology, School of Biological Sciences, Faculty of Biology, Medicine and Health, University of Manchester, Salford Royal Hospital, M6 8HD, Salford, UK; 2 Division of Neuroscience and Experimental Psychology, School of Biological Sciences, Faculty of Biology, Medicine and Health, University of Manchester, A V Hill Building, Manchester, M13 9PT UK; 3 Institute of Neurology, Queen Square Brain Bank for Neurological Disorders, Department of Molecular Neuroscience, University College London, 1 Wakefield St, London, WC1N 1PJ UK; 4 Cerebral Function Unit, Greater Manchester Neurosciences Centre, Salford Royal Hospital, Stott Lane, M6 8HD, Salford, UK

## Abstract

Frontotemporal Lobar Degeneration (FTLD) encompasses certain related neurodegenerative disorders which alter personality and cognition. Heterogeneous ribonuclear proteins (hnRNPs) maintain RNA metabolism and changes in their function may underpin the pathogenesis of FTLD. Immunostaining for hnRNP E2 was performed on sections of frontal and temporal cortex with hippocampus from 80 patients with FTLD, stratified by pathology into FTLD-tau and FTLD-TDP type A, B and C subtypes, and by genetics into patients with *C9orf72* expansions, *MAPT* or *GRN* mutations, or those with no known mutation, and on 10 healthy controls. Semi-quantitative analysis assessed hnRNP staining in frontal and temporal cortex, and in dentate gyrus (DG) of hippocampus, in the different pathology and genetic groups. We find that hnRNP E2 immunostaining detects the TDP-43 positive dystrophic neurites (DN) within frontal and temporal cortex, and the neuronal cytoplasmic inclusions (NCI) seen in DG granule cells, characteristic of patients with Semantic Dementia (SD) and type C TDP-43 pathology, but did not detect TDP-43 or tau inclusions in any of the other pathological or genetic variants of FTLD. Double immunofluorescence for hnRNP E2 and TDP-43 showed most TDP-43 immunopositive DN to contain hnRNP E2. Present findings indicate an association between TDP-43 and hnRNP E2 which might underlie the pathogenetic mechanism of this form of FTLD.

## Introduction

Frontotemporal Lobar Degeneration (FTLD) is a clinically, pathologically and genetically heterogeneous disorder affecting principally the frontal and temporal lobes of the brain. Three major clinical syndromes are recognised [34]. One syndrome, behavioural variant frontotemporal dementia (bvFTD), is characterised by changes in behaviour and personality and accounts for around 75% of all cases of FTLD, whereas the other two syndromes are disorders of language [34]. Semantic dementia (SD) (also known as semantic variant of primary progressive aphasia (svPPA)) is a disorder characterized by loss of conceptual knowledge of the meaning of words and objects [14, 34], whereas Progressive Non-Fluent Aphasia (PNFA) (also known as nfvPPA) is represented by an inability to construct language such that speech becomes hesitant and stuttering, becoming grammatically and contextually incorrect [14, 34]. The amyotrophic lateral sclerosis (ALS) form of motor neurone disease (MND) is seen in about 15% of patients with bvFTD, but is only rarely combined with either SD or PNFA [32].

Three different pathologies, characterised by abnormal neuronal, and sometimes glial, accumulations of aggregated proteins, are seen. Neuronal intracytoplasmic inclusions (NCI), composed of the microtubule associated protein tau, occur in about 45% cases as neurofibrillary tangle-like structures, or more rounded inclusions known as Pick bodies [33] and termed FTLD-tau [19]. The RNA and DNA binding protein, TDP-43, is present within NCI, neuritic processes (dystrophic neurites, DN) or neuronal intranuclear inclusions (NII) in about 50% of cases [2, 7, 26]. The relative proportions of NCI, DN and NII provide a neuropathological classification of FTLD-TDP subtypes [19]. FTLD-TDP subtype A is applied when NCI and short DN are both commonly present, mostly in outer cortical laminae, type B when NCI present throughout all cortical layers numerically predominate over DN, type C when long thick DN are present throughout all cortical layers and predominate over NCI and type D when NII are most common type of pathological change [19]. Most of the remaining 5% cases show NCI composed of the protein, Fused in Sarcoma (FUS), and are known as FTLD-FUS [19].

TDP-43 and FUS are heterogeneous nuclear riboproteins (hnRNP) [5, 30] and serve as RNA-splicing and transcription regulators, shuttling between nucleus and cytoplasm, thereby controlling cellular levels of protein synthesis. In the nucleus, TDP-43 binding encourages RNA stability, whereas in the cytoplasm it associates with stress granules and non-coding RNAs for post-transcriptional metabolism of RNA and transport. In FTLD-TDP there is a ‘clearing’ of normal physiological TDP-43 from the nucleus with its accumulation within the cytoplasm as NCI, DN or NII. However, the precise mechanism(s) directing this pathological change remain unclear.

Previous studies have pointed to specific interactions between particular hnRNPs and the pathological inclusions of FTLD. For example, we [9] and others [3, 23, 24] have shown that hnRNP A3 is present in the aggregates of dipeptide repeat proteins (DPR) in FTLD patients bearing expansions in *C9orf72* gene. Elsewhere, Gami-Patel and colleagues reported the presence of various hnRNPs, but especially hnRNP A1, within NCI in patients with the Neuronal Intermediate Filament Inclusion Body Disease form of FTLD-FUS [12].

TDP-43 is a stress responsive protein, and the TDP-43 aggregates in FTLD-TDP are thought to arise from stress granules [6, 18, 37]. Stress granules are transient cytoplasmic structures composed of mixed protein-RNA complexes, formed in response to cellular stress and believed to act as a sorting station, triaging mRNAs and sequestering transcripts not needed for coping with the stress [10]. Their composition and morphology varies according to the cell and stress type, but are generated by a reversible aggregation of prion-like core components such as the T-cell intracellular antigen-1 (TIA-1) and the poly A binding protein 1 (PABP1) [13] in order to regulate mRNA metabolism and protein translation [1, 18]. Biochemical studies have shown that TDP-43 associates with stress granules by interacting with TIA-1 [18]. Furthermore, using a yeast two-hybrid screen against TIA-1, hnRNP E2 was reported to be present in stress granules and processing bodies in HeLa cells, and an interaction between hnRNP E2 and TIA-1 was demonstrated by double immunofluorescence [11]. Finally, it has been reported that TDP-43 pathological inclusions in patients with bvFTD and FTLD-TDP pathology, and in others with ALS, co-localise with multiple markers of stress granules including TIA-1 [18]. Hence, it seems likely that both TDP-43 and hnRNP-E2 can be incorporated into stress granules under stress conditions.

With this in mind, we sought possible relationships between hnRNP E2 and the TDP-43 pathological inclusions of FTLD since, because of their shared links with stress granules, it is plausible that hnRNP E2 might be a component of the TDP-43 pathological inclusions that form within neuronal cell bodies (NCI) and processes (DN). We find that hnRNP E2 immunostaining co-localises with TDP-43 pathological changes, but only in patients with SD and type C TDP-43 histology. Collectively, present and previous [3, 9, 23, 24] data suggest that although pathological accumulations of TDP-43 or FUS are hallmark characteristics of FTLD, the fundamental biological mechanisms leading to these molecular end-points may differ between pathological subtypes.

## Materials and methods

### Patients

The study consisted of 2 groups comprising 90 subjects in total. One group had been recruited through Manchester Brain Bank (MBB), 54 with a clinical diagnosis of FTLD (30 males, 24 females; cases #1-54), and 10 healthy control subjects (3 males, 7 females; cases #55-64) (Table 1). The brains of these patients had been consecutively acquired by MBB over the years 1986 to present. All patients were from the North West of England and North Wales, and tissues were obtained through appropriate consenting procedures for the collection and use of the human brain tissues. All patients fulfilled relevant clinical diagnostic criteria [14, 25, 31], having been investigated longitudinally within a specialist dementia clinic using the Manchester Neuropsychological Profile (Man-NP) [35, 36] to determine and characterise the nature of their dementia.

**Table 1:** Selected clinical, neuropathological and genetic details on patients studied

case ID#	MRC ID#	clinical	TDP subtype	mutation	gender	PMI (h)	Onset (y)	Death (y)	Duration (y)
1*	BBN_5681	bvFTD	FTLD-TDP A	C9ORF72	M	74	49	58	9
2*	BBN_5706	bvFTD	FTLD-TDP A	C9ORF72	M	28	60	68	8
3*	BBN_5719	bvFTD	FTLD-TDP A	C9ORF72	F	74	59	64	5
4*	BBN_5752	bvFTD	FTLD-TDP A	C9ORF72	M	na	64	72	8
5	BBN_14793	bvFTD	FTLD-TDP A	C9ORF72	M	81	54	65	11
6	BBN_5756	FbvTD	FTLD-TDP B	C9ORF72	F	48	52	70	18
7*	BBN_5771	FTD + MND	FTLD-TDP B	C9ORF72	F	50	63	65	2
8*	BBN_5772	FTD + MND	FTLD-TDP B	C9ORF72	F	50	68	73	5
9	BBN_5691	FTD + MND	FTLD-TDP B	C9ORF72	M	72	60	62	2
10*	BBN_5663	FTD + MND	FTLD-TDP B	C9ORF72	M	36	57	59	2
11*	BBN_5742	PNFA	FTLD-TDP A	GRN V452WfsX38	M	57	66	71	5
12	BBN_10260	PNFA	FTLD-TDP A	GRN V452WfsX38	M	25	62	72	10
13*	BBN_5660	bvFTD	FTLD-TDP A	GRN V452WfsX38	F	13	53	71	18
14	BBN_5773	bvFTD	FTLD-TDP A	GRN Q130SfsX124	M	74	66	73	7
15	BBN_5715	PNFA	FTLD-TDP A	GRN Q130SfsX124	F	24	63	71	8
16*	BBN_5727	PNFA	FTLD-TDP A	GRN C31LfsX34	M	104	66	73	7
17	BBN_5718	bvFTD	FTLD-TDP A	GRN R493X	M	35	59	66	7
18	BBN_5675	bvFTD	FTLD-TDP A	GRNR493X	F	12	51	61	10
19	BBN_5686	bvFTD	FTLD-TDP A	GRN Q468X	F	48	60	66	6
20	BBN_5734	bvFTD	FTLD-TDP A	none	M	48	69	75	6
21*	BBN_5685	PNFA	FTLD-TDP A	none	M	19	68	78	10
22	BBN_5757	PNFA	FTLD-TDP A	none	F	72	66	77	11
23	BBN_5753	bvFTD	FTLD-TDP A	none	F	46	66	72	6
24	BBN005.28193	bvFTD	FTLD-TDP A	none	F	144	67	72	5
25	BBN005.29059	bvFTD	FTLD-TDP A	none	M	52	67	71	4
26	BBN_5661	FTD + MND	FTLD-TDP B	none	M	21	43	45	2
27*	BBN_5676	FTD + MND	FTLD-TDP B	none	M	na	60	68	8
28*	BBN_5701	FTD + MND	FTLD-TDP B	none	M	30	45	51	6
29*	BBN_5721	FTD + MND	FTLD-TDP B	none	M	87	58	69	11
30	BBN_5732	FTD + MND	FTLD-TDP B	none	F	164	50	52	3
31*	BBN_5764	FTD + MND	FTLD-TDP B	none	M	110	61	65	4
32	BBN_24314	FTD + MND	FTLD-TDP B	none	F	133	61	63	2
33	BBN005.28645	FTD + MND	FTLD-TDP B	none	M	114	64	69	5
34	BBN_5678	SD	FTLD-TDP C	none	M	48	54	68	14
35	BBN_5708	SD	FTLD-TDP C	none	F	28	55	66	11
36*	BBN_5720	SD	FTLD-TDP C	none	M	59	60	75	15
37	BBN_5726	SD	FTLD-TDP C	none	F	104	56	67	11
38	BBN_5736	SD	FTLD-TDP C	none	F	168	70	72	2
39*	BBN_5731	SD	FTLD-TDP C	none	M	44	71	77	6
40	BBN_16418	SD	FTLD-TDP C	none	M	29	56	68	12
41	BBN_19623	SD	FTLD-TDP C	none	F	86	68	82	14
42	BBN005.26066	SD	FTLD-TDP C	none	M	59	52	66	14
43	BBN005.28698	SD	FTLD-TDP C	none	M	35	57	77	20
44	BBN_5659	bvFTD	FTLD-tau	MAPT exon 10 + 16	M	46	50	61	11
45	BBN_5696	bvFTD	FTLD-tau	MAPT exon 10 + 16	F	52	46	58	12
46	BBN_5699	bvFTD	FTLD-tau	MAPT exon 10 + 16	M	30	43	55	12
47	BBN_5717	bvFTD	FTLD-tau	MAPT exon 10 + 16	F	23	52	65	13
48	BBN_5744	bvFTD	FTLD-tau	MAPT exon 10 + 16	F	33	50	60	10
49*	BBN_5733	bvFTD	FTLD-tau	MAPT exon 10 + 16	M	24	46	53	7
50	BBN_5760	bvFTD	FTLD-tau	MAPT exon 10 + 16	F	96	48	63	15
51	BBN_5763	bvFTD	FTLD-tau	MAPT exon 10 + 16	F	48	52	58	6
52	BBN_6081	bvFTD	FTLD-tau	MAPT exon 10 + 16	F	26	53	63	10
53	BBN005.29180	bvFTD	FTLD-tau	MAPT exon 10 + 16	F	83	60	69	9
54	BBN_5710	bvFTD	FTLD-tau	MAPT exon 10 + 13	M	43	65	70	5
55	BBN_3109	Control	none	none	F	72	na	76	na
56	BBN_3124	Control	none	none	F	44	na	82	na
57	BBN_3126	Control	none	none	M	52	na	80	na
58*	BBN_3378	Control	none	none	F	48	na	77	na
59	BBN_3447	Control	none	none	F	41	na	80	na
60	BBN_20608	Control	none	none	F	130	na	76	na
61	BBN_3337	Control	none	none	F	12	na	87	na
62*	BBN_3430	Control	none	none	M	49	na	84	na
63	BBN_25922	Control	none	none	F	103	na	100	na
64	BBN_25974	Control	none	none	M	93	na	91	na
65	BBN_8556	bvFTD	FTLD-TDP A	C9orf72	M	77	53	63	10
66	BBN_8109	PNFA	FLTD-TDP A	C9orf72	F	85	56	67	11
67	BBN_20082	PNFA	FTLD-TDP A	C9orf72	F	63	57	62	5
68	BBN007.29537	bvFTD	FTDL-TDP A	C9orf72	M	52	66	71	5
69	BBN007.26830	bvFTD	FTLD-TDP A	C9orf72	F	107	58	66	8
70	BBN_11628	bvFTD	FLTD-TDP A	TBK1	M	97	62	72	10
71	BBN_11698	bvFTD	FTLD-TDP A	none	F	85	57	63	6
72	BBN_11574	bvFTD	FTDL-TDP A	none	F	8	70	83	13
73	BBN_12507	bvFTD	FTLD-TDP A	none	M	93	57	62	5
74	BBN007.29538	bvFTD	FLTD-TDP A	GRN C31LfsX34	M	29	49	55	6
75	BBN_12169	bvFTD	FTLD-TDP B	C9orf72	F	94	64	66	2
76	BBN_12136	MND	FTLD-TDP B	none	M	70	67	69	2
77	BBN_11773	MND	FTLD-TDP B	none	F	30	75	77	2
78	BBN_12451	FTD/MND	FTLD-TDP B	none	F	46	63	67	4
79	BBN_12331	bvFTD	FTLD-TDP B	none	F	45	63	83	20
80	BBN_12480	SD	FTLD-TDP C	none	F	38	58	73	15
81	BBN_12524	SD	FTLD-TDP C	none	F	84	59	73	14
82	BBN_11594	SD	FTLD-TDP C	none	M	27	64	78	14
83	BBN_11883	SD	FTLD-TDP C	none	M	19	64	74	10
84	BBN_8614	SD	FTLD-TDP C	none	M	52	50	65	15
85	BBN_12300	SD	FTLD-TDP C	none	M	71	61	66	5
86	BBN007.26814	SD	FTLD-TDP C	none	M	76	44	67	23
87	BBN007.26851	PNFA	FTLD-TDP C	none	F	26	77	80	3
88	BBN007.26876	SD	FTLD-TDP C	none	F	31	58	72	14
89	BBN007.26890	SD	FTLD-TDP C	none	M	40	67	76	9
90	BBN007.29539	SD	FTLD-TDP C	none	F	25	52	65	13

*PMI* Post mortem delay interval, *na* data unavailable. Cases with asterix denote that new blocks were cut for the study from long-standing archived fixed tissues

The asterix (*) denotes cases where new tissue blocks were cut from archived samples, the original diagnostic samples no longer being available for analysis

The other group was recruited through Queens Square Brain Bank (QSBB) and comprised 26 patients with a clinical diagnosis of FTLD (12 males, 14 females; cases #65-90) (Table 1). The brains of these patients had been consecutively acquired by QSBB over the years 2004 to present. All patients were from London and South of England, and tissues were obtained through appropriate consenting procedures for the collection and use of the human brain tissues. Again, all patients fulfilled relevant clinical diagnostic criteria [14, 25, 31] having been referred to the Dementia Research Unit, Queen Square, London.

Of the combined 80 FTLD patients, 36 had been clinically diagnosed with bvFTD (17 males, 19 females; cases #1-6,13,14,17-20,23-25,44-54,65,68-75,79), 13 with bvFTD + MND or MND alone (9 males, 4 females; cases #7-10,26-33,76-78), 9 with PNFA (4 males, 5 females; cases #11,12,15,16,21,22,66,67,87) and 20 with SD (12 males, 8 females; cases #34-43, 80-86, 88-90) (Table 1). Pathologically, the FTLD group comprised 30 patients with FTLD-TDP type A (cases #1-5,11-25, 65-74), 18 with FTLD-TDP type B (cases #6-10,26-33,75-79), 21 with FTLD-TDP type C (cases #34-43,80-90), 11 with FTLD-tau (cases #44-54) (Table 1). Furthermore, within the FTLD group there were 16 patients with expansions in *C9orf72* (cases #1-10,65-69,75), 10 with *GRN* mutations (cases #11-19,74), 1 patient with *TBK1* mutation (case #70), 11 patients with intronic mutations in *MAPT* (cases #44-54) and 42 without known mutation (cases #20-43,71-73,76-90) (Table 1).

### Histological methods

Standard blocks of frontal (BA 8/9) and temporal (BA21/22) lobe, the latter to include the posterior hippocampus at the level of the geniculate bodies, were cut from the formalin fixed brains. Where possible, and in order to preserve maximum antigenicity, the original blocks taken for diagnosis were employed since these had been cut from brains which had been fixed in formalin for no more than 3-6 months from the time of acquisition. However, it was not possible to do this in all instances, especially in respect of some of the more long-standing cases (ie those acquired before 2007) where this block was no longer available and new blocks had to be cut from 20 cases for the purpose of this study (see Table 1 for those cases where new blocks were cut).

Paraffin sections were cut from these blocks at a thickness of 6 μm. Preliminary titration experiments were performed at dilutions 1:100 to 1:3000 on 5 randomly chosen cases, one from each pathological subgroup, in order to determine optimal specific nuclear and DN/NCI immunostaining for each antibody. When dilutions for hnRNP E2 staining less than what was optimal for nuclear and inclusion body staining were employed, immunostaining for nuclei and DN/NCI was still observed, but less strongly so, as would be expected given the lower antibody concentrations. Subsequently, all antibodies were employed at these optimised dilutions (as specified below) in a standard IHC protocol, as described previously [7–9, 22]. The following antibodies were employed: hnRNP E2 (also known as PCBP2) (mouse monoclonal, Santa Cruz, 23G: sc101136, 1:100 and Novus, mouse monoclonal H00005094-M07, 5F12 clone, 1:500), non-phosphorylated TDP-43 (rabbit polyclonal, 10,782-2-AP antibody, Proteintech, Manchester, UK, 1:3000) and phosphorylated TDP (pS409/410-2 antibody, Cosmo Biotech Ltd., Tokyo, Japan, 1:2000) and tau (mouse monoclonal, AT8, Innogenetics, Antwerp, Belgium, 1:750) proteins. Both the Santa Cruz and Novus hnRNP E2 antibodies are raised against recombinant hnRNP E2 of human origin, and on western blotting detect a protein with molecular mass of around 40 kDa. For all antibodies employed, antigen unmasking was performed by pressure cooking in citrate buffer (pH 6.0, 10 mM) over a 30-min period to include warming and cooling times, reaching 123 degrees Celsius for 30 s, and >15 psi pressure.

Double immunohistochemical staining was performed using phosphorylated TDP-43 antibody (pS409/410-2 antibody, Cosmo Biotech Ltd., Tokyo, Japan, 1:2000) and hnRNP E2 (Novus, 5F12, 1:500) antibody to investigate the co-localisation of the two proteins. Sections were cut, pre-treated and incubated in primary antibody as described above. Two Alexa Fluor secondary antibodies (Alexa Fluor 488 and Alexa Fluor 568; Molecular Probes, 1:300, with incubation for 1 hour at room temperature) were used to visualise sites of protein deposition. 4′-6-diamidino-2-phenylindol (DAPI) was used for nuclear counterstaining.

### Pathological assessment

Immunostained sections were examined microscopically for the appearance of intracellular distribution of staining within neurones of the temporal cortex, dentate gyrus and CA4 region of the hippocampus. These regions were chosen since it was known from previous work [7, 33] that the temporal cortex and dentate gyrus of the hippocampus are involved in all forms of FTLD-TDP or tau pathology in those patients with *MAPT* mutation [29]. Moreover, the CA4 region of the hippocampus was included because this is one of the principal regions affected by DPR pathology in patients with expansions in *C9orf72* [8, 22, 23]. The degree of neuronal nuclear and/or cytoplasmic hnRNP E2 immunostaining in each region was scored semi-quantitatively [9] at an objective magnification of ×25 (overall microscope magnification of ×250) employing the following rating scale:

- 0 = No staining present.
- 0.5 = rare (ie 1-5) cells per section showing weak nuclear and/or cytoplasmic staining.
- 1 = few (1-5) cells showing weak nuclear and/or cytoplasmic staining per ×250 microscope field.
- 2 = moderate number (5-10) cells showing moderate nuclear and/or cytoplasmic staining per ×250 microscope field.
- 3 = more than 10 cells showing strong nuclear and/or cytoplasmic staining per ×250 microscope field.

The severity of hnRNP E2-immunoreactive, and TDP-43-immunoreactive, inclusion body immunostaining (ie NCI and DN) in frontal and temporal cortex, and in granule cells of the dentate gyrus of the hippocampus, was separately graded at an objective magnification of ×25 (overall microscope magnification of ×250), employing the following rating scale:

- 0 = no inclusions present.
- 0.5 = rare (ie 1-5 inclusions per section).
- 1 = few (ie 1-5 inclusions per ×250 microscope field).
- 2 = moderate (ie 5-10 inclusions per ×250 microscope field).
- 3 = many (ie 10-50 inclusions per ×250 microscope field).
- 4 = very many (ie more than 50 inclusions per ×250 microscope field).

Scoring of staining was performed by a single observer (DMAM) blinded to clinical, histopathological and genetic status. Previous use of this particular scoring system has shown robust agreement in assessments when employed by both highly and lesser experienced observers [9].

Double immunolabelled sections were viewed with a Leica TCS4D confocal microscope using a 3-channel scan head and argon/krypton laser.

### Statistical analysis

Rating data was entered into an excel spreadsheet and analyzed using Statistical Package for Social Sciences (SPSS) software (version 17.0). The 80 FTLD patients were stratified according to genetic and pathological subtype for statistical analysis of the effect of each mutation and underlying pathology on the degree and pattern of hnRNP E2 staining. Comparisons of semi-quantitative scores for the intensity of neuronal hnRNP E2 immunostaining in nucleus and cytoplasm of neurones of the frontal and temporal cortex, and dentate gyrus of the hippocampus, with respect to pathological type or genetic mutation, were performed using Kruskal-Wallis test with post-hoc Mann-Whitney test where Kruskal-Wallis yielded a significant difference between antibody staining scores. Comparisons of semi-quantitative scores for the severity of hnRNP E2- and TDP-43-immunoreactive inclusions in frontal and temporal cortex, and dentate gyrus of the hippocampus, with respect to pathological subtype, were also performed using Kruskal-Wallis test. Comparison of scores for the severity of hnRNP E2- and TDP-43-immunoreactive inclusions in the same cases was made using Wilcoxon matched pairs test. Group comparisons of age at onset, age at death, post mortem delay interval and duration of illness were made using ANOVA with post hoc Tukey test. In all instances, significance levels were set at *p* < 0.05.

## Results

### Demographic comparisons

Comparison of the 4 FTLD pathology patient groups showed significant differences in mean age at onset of disease (F_3,76_ = 4.9, *p* = 0.004), mean age at death (F_3,76_ = 7.0, *p* < 0.001) and duration of illness did differ (F_3,76_ = 8.3, *p* < 0.001). Patients with FTLD-tau had an earlier age at onset than those with FTLD-TDP type A (*p* = 0.002), type B (*p* = 0.016) and type C (*p* = 0.012) pathology (Table 2) and an earlier age at death than those with FTLD-TDP type A (*p* = 0.014) and type C (*p* < 0.001) pathology, but not those with FTLD-TDP type B (*p* = 0.449) pathology. None of the 3 FTLD-TDP groups differed from each other in terms of age at onset, though patients with FTLD-TDP type B pathology died at an earlier age than those with FTLD-TDP type C pathology (*p* = 0.015). Patients with FTLD-TDP type B had a shorter disease duration than those with FTLD-TDP type C (*p* = 0.001) and those with FTLD-tau (*p* = 0.040); those with FTLD-TDP type A pathology also had a shorter disease duration than those with FTLD-TDP type C (*p* = 0.006), but there were no other differences between the other sub-types (Table 2). The healthy control group was also significantly older at death (*p* < 0.001) than each of the FTLD subgroups (Table 2).

**Table 2:** Mean +/− SD age at onset, death and duration of illness, and gender composition, for each neuropathological and genetic subgroup of patients. Mean +/− SD and median (in parentheses) post mortem delay interval (PMI) is also presented for each group

Group	M/F	Onset (y)	Death (y)	Duration (y)	PMI (h)
FTLD-TDP type A (*n* = 30)	17/13	60.7 ± 6.2	68.7 ± 6.1	8.0 ± 3.0	59.5 ± 33.3 (57)
FTLD-TDP type B (*n* = 18)	9/9	59.7 ± 8.0	65.2 ± 9.2	5.6 ± 5.5	70.6 ± 40.6 (50)
FTLD-TDP type C (*n* = 21)	12/9	59.7 ± 7.9	71.8 ± 5.3	12.1 ± 5.1	54.7 ± 35.1 (44)
FTLD-tau (*n* = 11)	4/7	51.4 ± 6.4	61.4 ± 5.4	10.0 ± 3.1	45.8 ± 24.0 (44)
FTLD *C9orf72* expansion (*n* = 16)	8/8	58.8 ± 5.3	65.7 ± 4.4	6.9 ± 4.6	66.1 ± 21.9 (72)
FTLD *GRN* mutation (*n* = 10)	6/4	59.5 ± 6.4	67.9 ± 5.9	8.4 ± 3.7	42.1 ± 29.2 (32)
FTLD No mutation (*n* = 42)	23/19	60.7 ± 7.9	70.0 ± 8.1	9.3 ± 5.5	62.6 ± 40.2 (48)
FTLD *MAPT* mutation (*n* = 11)	4/7	51.4 ± 6.4	61.4 ± 5.4	10.0 ± 3.1	45.8 ± 24.0 (44)
FTLD TBK1 mutation (*n* = 1)	1/0	62	72	10	97
Healthy Controls (*n* = 10)	3/7	na	83.3 ± 7.6	na	64.4 ± 35.0 (50.5)

Comparison of the 4 FTLD genetic patient groups also showed significant differences in mean age at onset of disease (F_3,75_ = 5.1, *p* = 0.003) and mean age at death (F_3,75_ = 5.0, *p* = 0.003), though duration of illness did not differ significantly (F_3,75_ = 1.2, *p* = 0.329). Patients with *MAPT* mutation had an earlier age at onset than those with *GRN* mutation (*p* = 0.050) and those without known mutation (*p* = 0.001), but not those with *C9orf72* expansion (*p* = 0.088). The other groups did not significantly differ from each other. Mean age at death was significantly earlier in *MAPT* than the no mutation group (*p* = 0.002), but otherwise there were no significant differences between all other groups (Table 2).

There were no significant differences between mean post mortem intervals for each pathological (F_4,83_ = 1.0, *p* = 0.401) or genetic (F_4,73_ = 1.6, *p* = 0.181) group (see Table 2), nor were there any significant correlations between each pathological measure and post mortem interval (*p* = 0.351-0.993).

### TDP-43 immunostaining

TDP-43- and tau-immunostaining was employed to classify the 80 FTLD patients into their respective pathological subgroups (FTLD-TDP subtypes A, B or C and FTLD-tau) according to the form and distribution of the TDP-43 or tau-immunoreactive inclusions (NCI, DN) present (see [19] for criteria). Median scores (with interquartile range) from TDP-43 immunostaining of pathological inclusions and neuronal nuclei and cytoplasm, derived from semi-quantitative scoring are shown in Table 3. As expected, given the inclusion of FTLD-tau group, the degree of TDP-43 inclusion body immunostaining (irrespective of whether these were in the form of NCI or DN, or both) differed between the 4 FTLD pathological groups in both the frontal (χ^2^ = 34.1, *p* < 0.001) and temporal (χ^2^ = 36.8, *p* < 0.001) cortex, and in the dentate gyrus (χ^2^ = 35.3, *p* < 0.001) with all 3 FTLD-TDP subtypes differing significantly from FTLD-tau group (*p* < 0.001 in every instance). However, there were no significant differences in the overall degree of TDP-43 immunostaining between FTLD-TDP type A, type B or type C subgroups.

**Table 3:** Median values with interquartile range for hnRNP E2 immunostaining of pathological inclusions, neuronal nuclei and cytoplasm in frontal and temporal cortex, and dentate gyrus of hippocampus, for each pathological subgroup

		Frontal cortex	
Group	E2 inclusions	E2 nucleus	E2 cytoplasm
FTLD-TDP type A	0 (0-0)	1 (0-1)	1 (0-2)
FTLD-TDP type B	0 (0-0)	0.75 (0-1.25)	1 (0-1.25)
FTLD-TDP type C	1 (1-2)	1 (0-3)	1 (0.5-2)
FTLD-tau	0 (0-0)	2 (1-2)	1 (0-2)
Healthy Controls	0 (0-0)	1.5 (0-3)	1.5 (0-2.25)
	Temporal cortex		
	E2 inclusions	E2 nucleus	E2 cytoplasm
FTLD-TDP type A	0 (0-0)	1 (0-1)	1 (0-2)
FTLD-TDP type B	0 (0-0)	0.75 (0-1.25)	1 (0-1.25)
FTLD-TDP type C	1 (1-3)	2 (0-3)	2 (0.5-3)
FTLD-tau	0 (0-0)	2 (1-2)	1 (0-2)
Healthy Controls	0 (0-0)	1.5 (0-3)	1.5 (0-2.25)
	Dentate Gyrus		
	E2 inclusions	E2 nucleus	E2 cytoplasm
FTLD-TDP type A	0 (0-0)	1 (0-1)	1 (0-2)
FTLD-TDP type B	0 (0-0)	0.75 (0-2)	1 (0-2)
FTLD-TDP type C	2 (0-3)	2 (0-3)	2 (0-3)
FTLD-tau	0 (0-0)	1 (0-3)	1 (0-2)
Healthy Controls	0 (0-0)	1.5 (0-3)	1.5 (0-2.25)
	TDP-43 inclusions		
	Frontal cortex	Temporal cortex	Dentate Gyrus
FTLD-TDP type A	3 (2-3)	3 (2-3)	1 (1-1.25)
FTLD-TDP type B	2 (2-3)	2 (2-3)	2 (1-3)
FTLD-TDP type C	2 (2-3)	3 (2-3)	3 (2-3)
FTLD-tau	0 (0-0)	0 (0-0)	0 (0-0)
Healthy Controls	0 (0-0)	0 (0-0)	0 (0-0)

### hnRNP E2 immunostaining

When using Santa Cruz hnRNP E2 antibody, 58/80 FTLD patients, and 8/10 controls, showed a variable level of cellular staining within nerve cells of the frontal and temporal cortex, and in granule cells of the dentate gyrus of hippocampus. When present, this was usually observed in both nucleus and cytoplasm, and ranged in intensity from very weak to strong. Conversely, 22 FTLD patients and 2 controls showed no nuclear or cytoplasmic staining at all in any region examined. Twenty of these cases had been stored for long periods (in excess of 10 years) in formalin fixation before blocks had been taken for this study, whereas the brains of those cases based on diagnostic blocks had been stored for a shorter time in formalin fixation before blocking (3-6 months), and all these, except 4 cases, showed variable degrees of nuclear and cytoplasmic staining. There did not appear to be any pathological or genetic group preference for presence/absence of this kind of staining. Hence, some degree of positive (nuclear or cytoplasmic, or both) staining was seen in 22/30 (73%) patients with FTLD-TDP type A histology, 11/18 (61%) patients with FTLD-TDP type B, 16/21 (76%) patients with FTLD-TDP type C, 9/11 (81%) patients with FTLD-tau and 8/10 (80%) controls. According to FTLD genetics, some positive degree of positive staining was seen in 9/16 (56%) patients with *C9orf72* expansion, 7/10 (70%) patients with *GRN* mutation, 32/42 (76%) patients with no mutation and 9/11 (80%) patients with *MAPT* mutation. There were no significant differences between the proportions of FTLD patients showing some degree of hnRNP E2 immunostaining when stratified either by pathological (χ^2^ = 2.09, *p* = 0.718) or genetic (χ^2^ = 1.98, *p* = 0.577) groupings.

Median scores (with interquartile range) from hnRNP E2 immunostaining of pathological inclusions and neuronal nuclei and cytoplasm, derived from semi-quantitative scoring, for each of the pathological groups are shown in Table 3. Statistical analysis showed no significant differences in scores for nuclear and cytoplasmic hnRNP E2 staining in frontal cortex (χ^2^ = 5.8, *p* = 0.120 and χ^2^ = 2.5, *p* = 0.479, respectively), temporal cortex (χ^2^ = 6.7, *p* = 0.080 and χ^2^ = 3.6, *p* = 0.312, respectively) or dentate gyrus (χ^2^ = 5.7, *p* = 0.128 and χ^2^ = 2.7, *p* = 0.444, respectively). Similarly, no significant differences in scores for nuclear and cytoplasmic hnRNP E2 staining in the 4 genetic groups were seen in frontal cortex (χ^2^ = 4.0, *p* = 0.400 and χ^2^ = 1.1, *p* = 0.891, respectively), temporal cortex (χ^2^ = 3.6, *p* = 0.463 and χ^2^ = 1.1, *p* = 0.889, respectively) or dentate gyrus (χ^2^ = 4.5, *p* = 0.338 and χ^2^ = 1.2, *p* = 0.882, respectively).

**Fig. 1:**
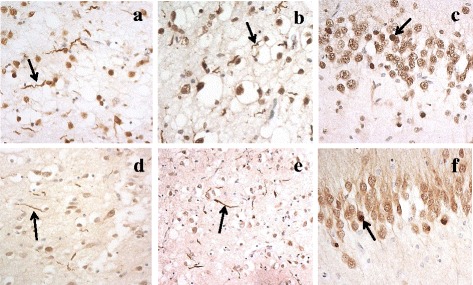
Dystrophic neurites seen on TDP-43 immunostaining (arrowed in **a** and **b**) were similarly seen on hnRNP E2 immunostaining (arrowed in d and e), in frontal (**a** and **d**) and temporal (**b** and **e**) cortex in FTLD-TDP type C cases. In the same cases rounded, solid-appearing NCI (arrowed) seen on TDP-43 immunostaining in dentate gyrus granule cells (**c**) were also seen in TDP-43 immunostaining (**f**). Immunoperoxidase-haematoxylin; microscope magnification ×400

DN with morphology akin to that seen on TDP-43 immunostaining (Fig. 1a and b) were seen on hnRNP E2 immunostaining (Fig. 1d and e) in the frontal (Fig. 1a and c) and temporal (Fig. 1b and e) cortex of 15/21 FTLD-TDP type C cases. In the same cases similar rounded, solid-appearing NCI seen on TDP-43 immunostaining in dentate gyrus granule cells (Fig. 1c) were also seen on hnRNP E2 immunostaining (Fig. 1f). Only FTLD-TDP type C cases had pathological inclusions labelled by hnRNP E2.There was no immunolabelling by hnRNP E2 of TDP-43 or tau inclusions in FTLD-TDP type A or type B cases or those with FTLD-tau. Interestingly, there was no apparent loss of physiological immunostaining of the nucleus for hnRNP E2 in those cells of the dentate gyrus bearing NCI (Fig. 1f), in contrast to that seen with TDP-43 where there was loss of normal nuclear staining in those cells containing TDP-43 immunoreactive NCI (Fig. 1c).

There were no clinical or neuropathological differences between the 15 hnRNP E2 positive cases (cases #37,40-43,81-90) and the 6 hnRNP E2 negative cases (cases #34-36,38,39,80). Moreover, there were no differences in age at onset (*p* = 0.542), age at death (*p* = 0.966), duration of illness (*p* = 0.405) or post mortem interval (*p* = 0.285) between the 15 positive and 6 negative cases. The only difference between the two groups was that the negative FTLD-TDP type C cases employed tissue blocks that had been newly cut for this study from long-standing (before 2007) archived fixed tissues as the original diagnostic blocks were no longer available for study. All other cases employed the original diagnostic blocks cut after 3-6 months fixation. Furthermore, within those FTLD cases bearing expansions in *C9orf72* gene, there was no immunostaining of the TDP-43-negative, p62-positive DPR inclusions within neurones of frontal and temporal cortex, or dentate gyrus granule cells and CA4 neurones of the hippocampus.

Consequently, semi-quantitative analysis within 5 pathological groups showed a significant difference in scores for hnRNP E2 positive inclusions in the frontal cortex (χ^2^ = 46.6, *p* < 0.001), temporal cortex (χ^2^ = 50.6, *p* < 0.001) and dentate gyrus (χ^2^ = 50.7, *p* < 0.001), with the number of inclusions in both areas being significantly different in FTLD-TDP type C cases than all other pathological subtypes (*p* < 0.001 in every instance). This result would be expected given the observations that only in FTLD-TDP type C cases were there pathological inclusions immunostained by hnRNP E2.

The results of immunostaining with the Novus hnRNP E2 antibody appeared broadly similar to those obtained with Santa Cruz antibody on all cases of FTLD-TDP type C, and selected cases of the other pathological subtypes, where both antibodies were employed, both with respect to the intensity of neuronal staining and severity of inclusion body staining. Comparison of semi-quantitative scores for the intensity of nuclear and cytoplasmic staining, and inclusion body staining, on all 21 cases of FTLD-TDP type C showed that Novus antibody immunostained nuclei less intensely than Santa Cruz antibody (frontal cortex *p* = 0.008, temporal cortex *p* = 0.005; dentate gyrus *p* = 0.005), but there were no significant differences between the antibodies for level of cytoplasmic staining (*p* = 0.617, *p* = 0.120 and *p* = 0.104, respectively). There were no significant differences between scores for inclusion body staining with both antibodies for frontal cortex (*p* = 0.054) and temporal cortex (*p* = 0.655) with only a trend for scores in dentate gyrus to be greater (*p* = 0.023) for Novus antibody.

**Fig. 2:**
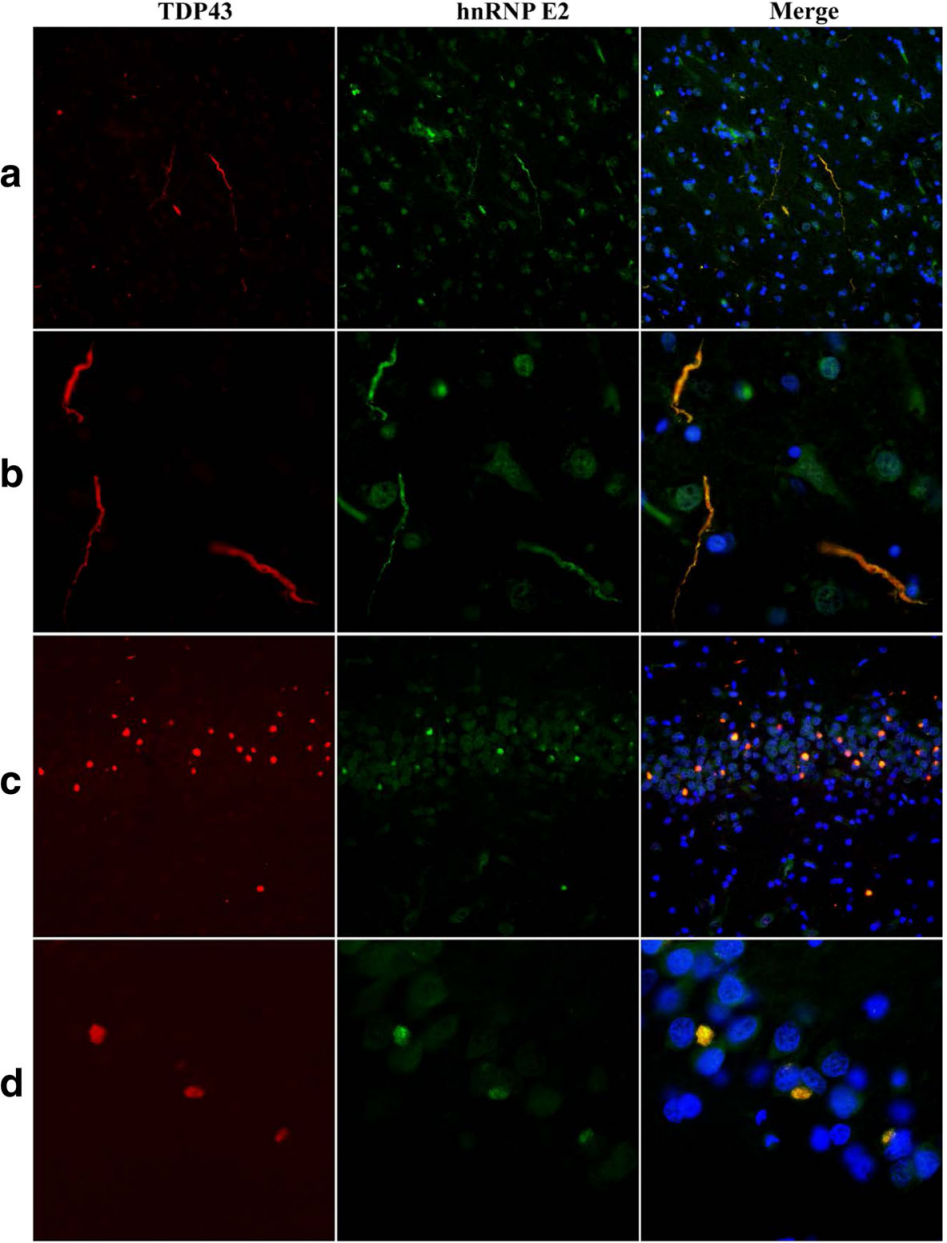
Immunofluorescence for TDP43 (*red*) and hnRNP E2 (*green*) in the temporal cortex (**a**,**b**) and dentate gyrus of the hippocampus (**c**,**d**) of FTLD cases. TDP43 and hnRNP E2 (merge) co-localize in both neuropil threads (**a**,**b**) and neuronal inclusions (**c**,**d**). Microscope magnification: ×200 (**a**,**c**); ×630 (**c**,**d**).

In general, the number of DN or NCI visualised on hnRNP E2 immunostaining appeared fewer than those seen in TDP-43 immunostaining (compare Fig. 1a with d, and b with e and c with f). Indeed, comparison of scores for TDP-43- and hnRNP E2- immunostaining of DN in FTLD-TDP type C cases showed that the number of DN immunostained for hnRNP E2 was significantly less than that immunostained for TDP-43 in frontal cortex (*p* = 0.004), temporal cortex *p* = 0.002) and dentate gyrus (*p* = 0.010). Consequently, double immunofluorescence labelling for TDP-43 and hnRNP E2 (using either the Santa Cruz or the Novus antibody) was performed on selected cases of FTLD-TDP type C and showed a good degree of colocalisation between the two proteins (Fig. 2).

## Discussion

In the present study, we have investigated the pattern of hnRNP E2 immunostaining across a range of clinical, pathological and genetic forms of FTLD. We found no significant changes in the amount, or intraneuronal distribution pattern, of neuronal hnRNP E2 immunostaining in any of the clinical, pathological or genetic FTLD subgroups. On the other hand, a strong immunostaining of DN and NCI, resembling that seen on TDP-43 immunostaining, was seen in 15/21 cases of SD with FTLD-TDP type C histology. No immunostaining of NCI, DN or NII for hnRNP E2 was seen in any of the other histological forms of FTLD-TDP, or in cases of FTLD-tau. However, it is not clear why only 15/21 cases of SD showed hnRNP E2-immunoreactive DN and NCI, despite there being plentiful TDP-43-immunoreactive DN and NCI present in the 6 other negatively staining cases. Long term storage of tissues in formalin rather than variations in clinical or pathological features, or differences in post mortem interval, is most likely responsible for the lack of hnRNP E2 immunostaining of DN and NCI, since it was necessary to prepare new samples from these 6 cases from archived fixed tissues as original freshly cut tissue blocks were no longer available for study. The complete lack of neuronal nuclear or cytoplasmic hnRNP E2 immunostaining in these same 6 cases supports this interpretation.

Interestingly, while incorporation of TDP-43 into NCI and DN was associated with loss of normal physiological TDP-43 immunostaining, there was no apparent loss of nuclear hnRNP E2 immunostaining in those cells of the dentate gyrus of the hippocampus containing hnRNP E2 immunoreactive NCI. Although, by analogy with TDP-43, this loss might have been anticipated to have occurred, this cannot be necessarily assumed to be the case, since while some hnRNP E2 protein will be binding to TDP inclusions during the formation/evolution of NCI (and DN), at the same time the remainder will still be available to participate in its normal physiological role (and generating nuclear staining) up until the time when the cell dies. It therefore cannot be assumed that all hnRNP E2 protein will be mislocalised into the cytoplasm, as is the case with TDP-43.

Present data therefore indicate an association between hnRNP E2 and TDP-43 pathology but only in this pathological form of FTLD. Indeed, double labelling immunofluorescence showed that most TDP-43 immunoreactive DN were immunoreactive for hnRNP E2 protein. In a previous study [9], we showed that DN and NCI in SD did not contain other hnRNPs such as hnRNP A1, A2/B1 or A3 suggesting that incorporation of hnRNP E2 into DN is not simply due to a passive recruitment of hnRNP E2 protein, along with other hnRNPs, into the aggregating protein conglomerate. It is not clear why co-localization was not complete, but this again could be due to technical reasons such as antigen preservation or accessibility. Alternatively, it might indicate that hnRNP E2 protein is incorporated into pre-existing TDP-43 pathological inclusions at later points in time, with those inclusions that are hnRNP E2 negative not having had sufficient time to accrue enough hnRNP E2 protein to be detectable by immunohistochemistry.

The results of this study imply an important role for hnRNP E2 in the pathogenesis of SD. hnRNP E1 and E2 are the most highly expressed and well characterised isoforms of hnRNP E proteins in human tissues with 89% amino acid homology. They belong to the hnRNP K protein family and have a triple hnRNP K (KH) domain, designated KH1, KH2 and KH3, through which they can bind both poly(rC) regions and low rC mRNAs [20, 27, 28]. They can shuttle between nucleus and cytoplasm and participate in the regulation of mRNA stability and translation [20, 27]. It is believed that hnRNP E1 is encoded by an intronless gene that is a product of a retrotransposition event of a fully processed minor isoform of hnRNP E2 [21]. Each of the KH domains is able to interact independently with a target RNA sequence which gives this protein a potentially high number of complex specific RNA interactions. Woolaway and colleagues demonstrated that depletion of either hnRNP E1 or hnRNP E2 lead to increased production of HIV-1 structural proteins, whereas overexpression of hnRNP E1, but not hnRNP E2, inhibited expression of Rev.-dependent RNAs encoding gp120 and p24 [38]. Other work has demonstrated a higher affinity of hnRNP E1 for hnRNP D than hnRNP E2 [17], and hnRNP E1 and hnRNP E2 have differential responses to hypoxic stress [39]. Both hnRNP E1 and hnRNP E2 can regulate BC200 RNA-mediated translation inhibition but not through the same control mechanism [16]. Therefore, despite the high degree of sequence similarities between hnRNP E1 and hnRNP E2 isoforms, they each have distinct non-redundant cellular functions.

Little is known concerning any specific role for hnRNP E2 within the nervous system in health or neurodegenerative disease, beyond that of control of mRNA stability and mRNA translation. Broderick and coworkers reported that hnRNP E2 can bind to exon 10 of *MAPT* and activate/regulate alternative splicing [4]. Mis-splicing of exon 10 is a cause of that form of FTLD-tau, known as Frontotemporal Dementia with parkinsonism linked to chromosome 17 (FTDP-17) in which there is increased use of this splice site leading to an imbalance in the ratio of 3-repeat and 4-repeat tau isoforms, in favour of 4-repeat tau, with aggregation of the excess 4-repeat tau into neurofibrillary tangle-like structures [15, 29]. In the present study we investigated 11 cases of FTDP-17 associated with intronic mutations affecting exon 10, but did not find any changes in neuronal hnRNP E2 immunoreactivity or binding of hnRNP E2 to aggregated tau, implying that the functional disturbances leading to increased splicing of exon 10 in this form of FTLD are not mediated by changes in hnRNP E2. Such a conclusion would be supported by the lack of tauopathy in patients with SD where hnRNP E2 pathological changes are associated with TDP-43 proteinopathy instead.

To our knowledge, there have been no previous reports documenting any direct functional or pathological association between hnRNP E2 protein and TDP-43, and the mechanism leading to binding of hnRNP E2 to DN and NCI in patients with SD remains unclear. It is plausible that this scenario could underpin the presence of hnRNP E2 and TDP-43 in DN in patients with SD. However, it is not clear why, in the present report, hnRNP E2 was not also seen in the TDP-43 pathological inclusions of other forms of FTLD-TDP (ie FTLD-TDP types A and B) when these have also been shown to contain markers of stress granules such as TIA-1 [18].The absence of hnRNP E2 in the TDP-43 pathological inclusions of other forms of FTLD-TDP points to a disease mechanism which is specific to patients with SD and FTLD-TDP type C pathology, and one not shared by other forms of FTLD-TDP despite all pathological forms being linked by the same pathological ‘end product’.

## Conclusions

In the present study we have shown that a high proportion of TDP-43-positive DN in patients with SD contain hnRNP E2 protein; no other histological forms of FTLD-TDP showed this association, nor were NCI in FTLD-tau hnRNP E2-immunoreactive despite evidence that hnRNP E2 may function as a modulator of alternative splicing of *MAPT*. The association between hnRNP E2 and TDP-43 in DN in SD, when taken in conjunction with previous findings showing specific interactions between hnRNP A1 and FUS-positive NCI [12] and hnRNP A3 and DPR in *C9orf72* expansion carriers [3, 9, 23, 24], suggests that specific changes in different hnRNPs might underlie each pathological form of FTLD. The exact nature of how these proteins (hnRNP E2, TDP) might interact is outside the scope of the present study and requires further work, employing expression studies, western blotting or pull-down methodologies, for example, to support the present argument that an increase in hnRNP E2 protein in NCI is specific to FTLD-TDP type C.
